# Generation and Characterization of an Anti-diclazuril Monoclonal Antibody and Development of a Diagnostic Enzyme-Linked Immunosorbent Assay for Poultry

**DOI:** 10.3389/fnut.2022.910876

**Published:** 2022-05-16

**Authors:** Hong Shen, Chao Li, Han Sun, Wanqin Chen, Bilian Chen, Yu Yi, Jianfeng Mei, Yanlu Zhang, Guoqing Ying

**Affiliations:** ^1^College of Pharmaceutical Science, Zhejiang University of Technology, Hangzhou, China; ^2^Department of Biological Inspection, Zhejiang Institute for Food and Drug Control, Hangzhou, China

**Keywords:** drug residue, identification, coccidiosis, variable region, food safety

## Abstract

An anti-diclazuril monoclonal antibody (mAb) was developed for use in enzyme-linked immunosorbent assay (ELISA)-based detection of diclazuril with high sensitivity and specificity, which can be used to measure anti-coccidial drug residues. The anti-diclazuril mAb had a half-maximal inhibitory concentration of 0.449–0.517 ng/mL. The mAb cross-reactivity with toltrazuril, toltrazuril 18 sulfone, clozaril, monesin, madurmycin, and salinomycin was very minimal (< 0.1%). The detection limit of the ELISA using this mAb was 0.10 ng/mL and the sensitivity was 0.05 ng/mL. A standard curve generated in the range of 0.05–16.2 ng/mL had a linear correlation coefficient value of ≥ 0.99. The average recoveries of diclazuril from chicken and duck samples ranged from 85.0 to 102.5%.Intra- and inter-assay coefficients of variation ranged from 5.9 to 8.5% and 9.2 to 12.6%, respectively. Using the International Immunogenetics Information System^®^, the VH domain of the mAb was found to be encoded by an IGHV3 family gene and had the following complementarity determining region (CDR) sequences: GFTFSRY (CDR1), SRGGS (CDR2), and GDDNYAFAY (CDR3). The VL domain was encoded by an IGKV1 family gene and had the following CDR sequences: KSSQSLLNSRTRKNYLA (CDR1), WASTRES (CDR2), and KQSYNLHT (CDR3). This study provides a method to generate anti-diclazuril mAbs and determine their variable region sequences. The diagnostic ELISA developed using this mAb may drive additional studies on the monitoring and detection of food and veterinary drug residues.

## Introduction

Coccidiosis is the most common epidemic parasitic disease in the intensive breeding industry (1, 2). Most livestock are highly susceptible to infection and the disease results in high mortality. Long-term drug administration is the only method to prevent and control coccidiosis in farm animals (3–5). Diclazuril, a triazine acetonitrile compound, is a non-polyether synthetic anti-coccidial drug that was developed by Janssen in the 1980s (6, 7). It is a highly efficient, low toxic, and broad-spectrum anti-coccidiosis drug. Diclazuril effectively reduces economic losses from coccidiosis in the poultry industry and is widely used in animal breeding to prevent and manage coccidiosis (8–10). Despite these benefits, diclazuril and similar drugs may leave residues in some animal-derived foods, which can cause problems in terms of food safety (11–13). The Joint Expert Committee on Food Additives and the European Medicines Agency evaluated data from toxicological studies of diclazuril and recommended acceptable daily intakes and maximum residue limits (14). Diclazuril is a coccidiostat currently authorized as a feed additive in the European Union with a legal limit of 1 mg/kg. In sheep and rabbit meat, liver, kidneys, and crude fat, the legal limits are 0.5, 3, 2, and 1 mg/kg, respectively. In other agricultural species, the limits in the liver, kidneys, and other meat products are 0.04, 0.04, and 0.005 mg/kg, respectively. The Chinese GB 31650-2019 Veterinary Drug Residue Limits Standard stipulates that the maximum residue limit of diclazuril in poultry, which is banned during egg laying, is 0.5 mg/kg in muscle. The maximum residue limit of diclazuril has also been stipulated in Announcement No. 2002-235 from the Ministry of Agriculture, China. In recent years, methods such as high-performance liquid chromatography (15, 16), gas chromatography, and liquid chromatography tandem mass spectrometry (17–19) have been widely used to detect diclazuril residues. However, these methods are unsuitable for on-site spot, rapid, and batch inspections by enterprises and governments. Thus, it remains difficult to meet the current need for rapid diclazuril detection. Enzyme-linked immunosorbent assays (ELISAs), which have the advantages of high throughput, high efficiency, and low cost, have been gradually applied to rapid quantitative detection of various veterinary drug residues in animal-derived foods (20, 21). ELISAs avoid the need for complex preprocessing of samples, greatly increase throughput, do not require the use of expensive instruments, and are easy to interpret (22–24). Wang et al. (25) developed a colloidal gold immunoassay for diclazuril with a detection limit of 2 μg/kg. Li et al. (15) reported a highly efficient chemiluminescent immunoassay to detect diclazuril in chicken muscle with a half maximal inhibitory concentration (IC_50_) of 0.48 μg/kg. The limit of detection for diclazuril was 0.02 μg/kg. Zhang et al. (26) developed a detection method for diclazuril in chickens by employing a one-step ELISA with an IC_50_ value of 0.952 μg/kg. The immunoassay method has the advantages of simple development, low cost, simple operation, and rapid detection, which can achieve high-throughput rapid screening, and is widely used in livestock and poultry farms. However, it cannot achieve simultaneous quantitative detection of various residual drugs. A successful immunoassay must have three elements: Stable antibodies, sensitive and specific markers, and efficient separation methods. The preparation of traditional monoclonal antibodies is tedious, which has become a limiting factor in immunoassay development (27). Therefore, in this study, an anti-diclazuril antibody with high sensitivity, specificity, and stability was obtained by hapten modification. Furthermore, the variable region gene sequence of the antibody was obtained by gene sequencing the variable region of the antibody recognition site. This study provides a feasible method for batch preparation of stable antibodies through a cell expression system.

## Materials and Methods

### Materials

BALB/c mice (license number: SCXK2015-0018) were purchased from Hubei Provincial Laboratory Animal Research Centre (Wuhan, China). Dixiezhu and toltrazuril standards were provided by Dr. Ehrenstorfer GmbH (Augsburg, Germany). Complete and incomplete Freund’s adjuvant, ELISA coating buffer (20×), phosphate-buffered saline (PBS; 10 ×), Tris-buffered saline containing 0.1% (v/v) Tween-20 washing solution (10×), blocking solution with bovine serum albumin (BSA), an EL-Tetramethylbenzidine Chromogenic Reagent Kit, ELISA stop buffer, and enzyme-labeled secondary antibody (horseradish peroxidase-conjugated goat anti-mouse IgG) were all obtained from Shanghai Shenggong (Shanghai, China). Hypoxanthine-thymidine and hypoxanthine-aminopterin-thymidine (HAT) media were purchased from Sigma (St. Louis, MO, United States). A Mouse monoclonal antibody (mAb) isotyping kit was purchased from Sino Biological (Beijing, China), an RNAeasy Mini Kit (74106) was obtained from Qiagen (Dusseldorf, Germany), and the SuperScript^®^ III First-Strand Synthesis System for RT-PCR (18080-051) was purchased from Invitrogen (Carlsbad, CA, United States). A Gel DNA Purification Kit (GK2042) was purchased from Generay (Shanghai, China) and a Plasmid miniprep kit (PD1211-02) was purchased from Biomega (Salt Lake City, Utah, United States).

### Synthesis of Diclazuril Derivatives

Diclazuril (204 mg) was dissolved in 10 mL dimethyl formamide (DMF). Then, 110 mg of carboxymethyl hydroxylamine was weighed and dissolved in 2 mL methanol followed by 100 μL pyrrolidine. The carboxymethyl hydroxylamine solution was then added to the diclazuril solution and the reaction was stirred at 50°C. Reaction progress was monitored by thin layer chromatography. Once the reaction had completed, the mixture was removed and dried in a vacuum. The spin-dried substance was later reconstituted in 10 mL H_2_O and the pH was adjusted with 1 M hydrochloric acid to pH 2, after which a white precipitate appeared. Next, 20 mL of ethyl acetate was added (two extractions with 10 mL each) and the organic phase was obtained. Then, 20 mL of 1 M NaOH was added (10 mL at a time) to the organic phase to merge the aqueous phases. The pH was adjusted to 2 using hydrochloric acid in an ice bath until a white precipitate had appeared. This solution was extracted three times with ethyl acetate and the organic phase was merged. The organic phase was washed with H_2_O to neutrality. After adding an appropriate amount of anhydrous sodium sulfate, the mixture was dried in a vacuum and stored at 4°C until use.

### Diclazuril Holoantigen Synthesis

To generate diclazuril-BSA, 10 mg diclazuril carboxyl derivative, 10 mg dicyclohexylcarbodiimide, and 6 mg N-hydroxysuccinimide were dissolved in 0.5 mL DMF and stirred overnight at 4°C. After centrifugation of this mixture, the supernatant was collected as solution A. To obtain solution B, 32 mg BSA was dissolved in 10 mL of 1 × PBS. Solution A was then mixed with solution B, followed by stirring for 8 h at 4°C, after which the solution was collected and dialyzed against 1 × PBS for 2 days. To generate diclazuril-ovalbumin (OVA), 10 mg diclazuril carboxyl derivative, 10 mg dicyclohexylcarbodiimide, and 6 mg N-hydroxysuccinimide were dissolved in 0.5 mL DMF and then stirred at 4°C overnight. After centrifugation of this mixture, the resulting supernatant was collected as solution A. To obtain solution B, 54 mg OVA was dissolved in 10 mL of 1 × PBS. Solution A was then mixed with solution B and stirred for 8 h at 4°C. Subsequently, the solution was collected and dialyzed against 1 × PBS for 2 days. Then, the coupling ratio of the final immunogen was calculated.

### Hybridoma Cell Preparation

Three healthy 6–8-week-old female BALB/c mice were immunized with the diclazuril-BSA conjugate. Blood was collected after three immunizations and the polyclonal serum antibodies reacted with a standard concentration of 50 ng/mL diclazuril in a competitive reaction format. A western blot with sera from the BALB/c mice was used for fusion to produce positive results. Three days before the fusion, an intraperitoneal booster immunization with the adjuvant-free antigen was administered to each mouse at a dose of 100 μg. Three days after the booster immunization, mouse blood was collected from the orbital vein and centrifuged to obtain positive control serum. Approximately 1 × 10^8^spleen cells were collected under aseptic conditions and placed at room temperature. Next, 30 mL incomplete medium was added, the mixture was centrifuged, and the resulting supernatant was discarded. To loosen the cell pellet, the bottom of the tube was gently inverted and then 1 mL pre-warmed polyethylene glycol fusion agent was slowly added to the cells along the tube wall while rotating the centrifuge tube and stirring for 60 s. To dilute the polyethylene glycol, 20 mL incomplete medium was added immediately. After centrifugation, the supernatant was discarded and complete medium with HAT was added to prepare a cell suspension. The cells were then cultured in a 96-well cell culture plate in an incubator at 37°C with a humidified atmosphere that contained 5% CO_2_. Because the cells were cultured in HAT medium, unfused myeloma cells and unfused lymphocytes died gradually, while fused hybridoma cells survived and proliferated in the HAT medium. After 7–10 days of culture, an ELISA plate was coated with diclazuril-OVA conjugate at 37°C for 2 h and then blocked with 2% (w/v) BSA. The supernatants of clonal hybridoma cells grown in the 96-well plate were pipetted onto the BSA-blocked ELISA plate that was incubated at 37°C for 1 h. After washing five times, horseradish peroxidase-labeled goat anti-mouse IgG was added, followed by incubation for another 1 h at 37°C. After additional washes, tetramethylene benzidine substrate was added to each well and the reaction was terminated with 2 M sulfuric acid. The optical densities of the wells were recorded using a microplate reader. Clones whose supernatants yielded positive ELISA signals were picked for expansion in a 24-well cell culture plate. After 3 days of additional culture, another ELISA screening was performed as described above. The selected ELISA-positive clones were subjected to western blot rescreening. Cells that yielded positive western blot results were subcloned twice and monoclonal cells capable of stably secreting antibodies were selected for further use. Following additional amplification in cell culture, these clones were frozen and stored.

### Antibody Purification From Ascites

Ascites antibodies were prepared by *in vivo* induction. BALB/c mice (6–8 weeks old) were intraperitoneally injected with 0.5 mL sterile paraffin oil. Hybridoma cells (5 × 10^5^) were injected intraperitoneally 7 days later and ascites were collected 7 days thereafter. The anti-diclazuril mAb was purified by the octanoic acid-saturated ammonium sulfate method (22).

### Sample Pretreatment

Chicken and duck samples were obtained from a local market and confirmed to be negative for diclazuril by HPLC-MS/MS.

To prepare positive samples, six confirmed negative chicken and duck samples were mixed with diclazuril standard at 0.2, 0.4, and 0.8 μg/kg. Each concentration had three replicates, and three batches were used. Each sample was extracted in accordance with the following method. Ground chicken and duck tissues (1.0 ± 0.05 g) were mixed with 1 mL of 0.2% phosphoric acid and 3 mL acetonitrile for 2 min and then centrifuged (4,000 *g*) at room temperature for 5 min. Then, 1 mL of the upper organic phase was transferred to a glass tube and dried in a 65°C water bath under nitrogen flow. Sample diluent (1 mL) was added, followed by vortexing for 30 s, and then 50 μL was collected for analysis.

### Design of the Diagnostic Enzyme-Linked Immunosorbent Assay

Isotyping of mAbs was carried out using a mouse monoclonal antibody isotyping kit. The optimal paired antibodies and antigens were screened from ascites by the chessboard method. Through design optimization of the coating antigen concentration, monoclonal antibody diluent, incubation time, standard curve range, and sample pretreatment steps, we developed the indirect competitive ELISA method for diclazuril detection.

Diclazuril standard or a pre-treated sample at 50 μL/well was added to a microwell of the enzyme plate coated with the diclazuril-coupling antigen. Then, a horseradish peroxidase-labeled sheep anti-mouse antibody was added at 50 μL/well, followed by 50 μL/well anti-diclazuril monoclonal antibody working solution. After gentle shaking to mix, the plate was allowed to react for 30 min at 25°C. After discarding the supernatant, the plates were washed four times with PBS (pH 7.4). Then, 50 μL carbamide peroxide and 50 μL tetramethylbenzidine were added to each well to react for 15 min in the dark. The enzymatic reaction was stopped by adding 50 μL/well of 2 M sulfuric acid. The optical density value of each well was then determined at 450 nm.

A standard curve was constructed with inhibition as the ordinate and the logarithm of the standard concentration as the abscissa. The sensitivity and linear range of the established method were obtained by the standard curve. Twenty negative chicken and duck samples were analyzed, and the concentration corresponding to each absorbance was determined using the standard curve. The detection limit was determined by the average concentration of 20 samples plus three times the standard deviation.

Antibody specificity was evaluated by comparing the ability of the mAb to bind to diclazuril as well as toltrazuril, toltrazuril sulfone, clozaril, monensin, madurmycin, and salinomycin. The analogous molecules were diluted and then indirectly competed with the mAb in the ELISA. A standard curve was produced and IC_50_ values were calculated. Cross-reactivity was calculated as follows: (diclazuril concentration that causes 50% inhibition/concentrations of other anticoccidial drugs that cause 50% inhibition) × 100%. For immunoassays, spiked recovery experiments are often performed to assess the accuracy of the method. Because diclazuril is mainly used as a feed additive in poultry farming, different types of chicken and duck samples were used to determine spiked recovery of the anti-diclazuril mAb. Three batches of antibodies were prepared from the same cell line, three batches of ELISA kits were prepared, and three parallel tests were carried out to verify the variation between batches.

The optical density values of supernatant samples were measured using an indirect competitive ELISA to calculate the inhibition rate, which was then compared with the standard curve regression equation to calculate the spiked recovery in accordance with the following formula: [spiked recovery (%) = spiked value (ng/g)/measured value (ng/g)] × 100%.

### Comparison With the Instrument Method

Ten confirmed negative chicken and duck samples and 10 chicken and duck samples with 10 ng/mL diclazuril were used. The coincidence rate of positive samples was determined by the ELISA method and the current instrument method, namely HPLC-MS/MS. The instrument testing method referred to SN/T2318-2009 (28).

### Cloning and Sequencing of Heavy and Light Chain Variable Region Genes of the Anti-diclazuril mAb

A hybridoma clone that secreted an anti-diclazuril mAb with both high affinity and specificity was identified. Total RNA was extracted using the RNeasy Mini Kit and quantified using a NanoDrop spectrophotometer. The RNA was assessed by 1% non-denaturing agarose gel electrophoresis and then reverse transcribed into cDNA using the SuperScript^®^ III First-Strand Synthesis System for RT-PCR. The genes that encoded the VL and VH domains of the anti-diclazuril mAb were amplified using specific primers. The primers are listed in Table 1. The corresponding PCR amplicons were then subjected to 1% agarose gel electrophoresis and the target fragment was obtained by gelatinization. After purifying the desired product using the Gel Recovery Kit (GK2042), the purity of the target fragment was assessed by 1% agarose gel electrophoresis. The corresponding VH and VL fragments were cloned into the sequencing vector PUC57. After VH and VL were sequenced by Genecreate Co., Ltd. (Wuhan, China) using a 3730 DNA Analyzer (ABI, CA, United States), homology and structural analyses were performed using IMGT/V-QUEST. To amplify VH of the anti-diclazuril antibody, 5’ and 3’ degenerate primers for the antibody light and heavy chains were synthesized on the basis of the FR region sequence (for detailed experimental procedures, see Supplementary Material).

**TABLE 1:** Degenerate primers for antibody light and heavy chains.

Chain region	Primer sequences
VH(5′–3′)	GATGTGAAGCTTCAGGAGTC CAGGTGCAGCTGAAGGAGTC CAGGTGCAGCTGAAGCAGTC CAGGTTACTCTGAAAGAGTC GAGGTCCAGCTGCAACAATCT GAGGTCCAGCTGCAGCAGTC CAGGTCCAACTGCAGCAGCCT
VH (3′–5′)	TGCAGAGACAGTGACCAGAGT TGAGGAGACTGTGAGAGTGGT TGAGGAGACGGTGACTGAGGT TGAGGAGACGGTGACCGTGGT
VL(5′–3′)-Kappa	GATGTTTTGATGACCCAAACT GATATTGTGATGACGCAGGCT GATATTGTGATAACCCAG GACATTGTGCTGACCCAATCT GACATTGTGATGACCCAGTCT GATATTGTGCTAACTCAGTCT GATATCCAGATGACTCAGTCT GACATCCAGCTGACTCAGTCT CAAATTGTTCTCACCCAGTCT
VL(3′–5′)-Kappa	CCGTTTCAGCTCCAGCTTG CCGTTTTATTTCCAGCTTGGT CCGTTTTATTTCCAACTTTG GGATACAGTTGGTGCAGCATC

## Results and Discussion

### Haptens and Conjugates

**FIGURE 1:**
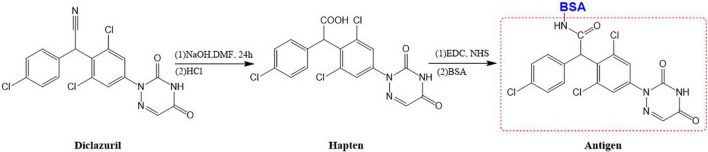
Transformation path of diclazuril hapten.

The purpose of hapten design is to transform its structure so that it has a group of couplable carrier proteins because a hapten has only reactivity and no immunogenicity. When a hapten has a group of couplable carrier proteins, it becomes a complete antigen and has both reactivity and immunogenicity. Therefore, when designing the hapten by computer simulation, it had to be as similar as possible to diclazuril in terms of molecular structure, spatial conformation, and electron distribution, and the carbon chain length of the designed connecting arm should be between 3 and 6. There should also be active groups that can be coupled to protein carriers. The conventional methods of hapten synthesis are diazotization and CMO derivatization. However, diclazuril is a small molecular amide compound. Therefore, the conventional experimental scheme was unsuitable. Thus, a hapten modification scheme that introduced a COOH active group was designed by computer simulation. The hapten was synthesized by introducing a carboxyl group into aldehydes and ketones under acidic or alkaline conditions. Finally, the carrier protein BSA/OVA was coupled to form a complete antigen. An immune reaction in mice demonstrated successful preparation of the hapten. Figure 1 shows the transformation process of the diclazuril hapten.

### Characterization of Diclazuril Conjugates by Ultraviolet Scanning Spectroscopy

**FIGURE 2:**
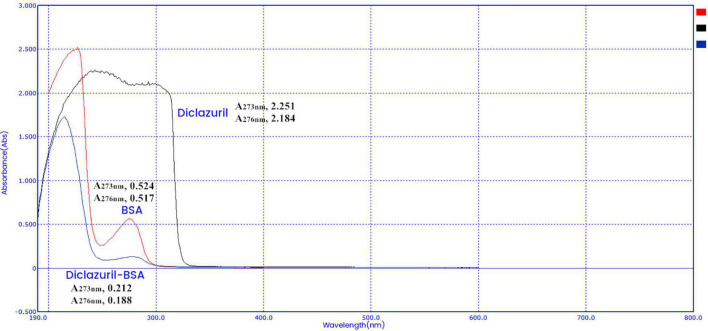
UV scanning spectroscopy of the diclazuril-bovine serum albumin (BSA) conjugate.

**FIGURE 3:**
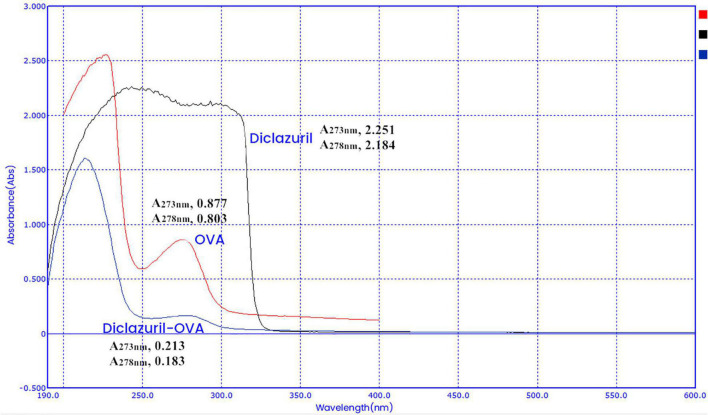
UV scanning spectroscopy of the diclazuril-ovalbumin (OVA) conjugate.

Diclazuril conjugates were characterized by full-wavelength ultraviolet scanning spectroscopy (Figures 2, 3). The maximum absorption wavelengths of diclazuril were 228 nm and 273 nm. The maximum absorption wavelengths of diclazuril-BSA and BSA were 275 nm and 276 nm, respectively. The maximum absorption wavelengths of diclazuril-OVA and OVA were 275 and 278 nm, respectively. The maximum absorption wavelength changed slightly after hapten coupling with each carrier protein, which indicated successful synthesis of both conjugates. The coupling ratio of the complete antigen was calculated by the formula:


$$C\,\text{a}/C\,\text{b}=\frac{\left(A\,C\,\mathrm{am}*K\,B\,\mathrm{bm}-\mathrm{ACbm}*\mathrm{KBam}\right)}{\left(\mathrm{ACbm}*\mathrm{KAam}-\mathrm{ACam}*\mathrm{KAbm}\right)}$$

Aa, Bb, and Cc refer to small molecule drugs, carrier proteins, and conjugates, respectively. AAam, ABam, and ACam refer to the absorbances of these three substances at the maximum characteristic absorption wavelengths of small molecule drugs. AAbm, ABbm, and ACbm refer to the absorbances of these three substances at the maximum characteristic absorption wavelengths of carrier proteins, respectively. KAxm = AAxm / (Px/Mx) (P is the concentration, M is the molecular weight, x represents a, b, and c). The calculated coupling ratio of diclazuril-BSA was 19 and that of diclazuril-OVA was 12.

### Identification of the Anti-diclazuril Monoclonal Antibody and Cross-Reactivity

Three mice were immunized with the immunogen. After three immunizations and one booster immunization, five positive cell lines were obtained after fusion cloning. The subtypes of the five cell lines were identified. The four cell lines—7B3 5F7, 3G7 2H10, 2B7 1A7, and 6G10 5F4—were all IgG1, and the 3D6 2E4 cell line was IgG3. Supernatant analysis showed that the titer of the 3D6 2E4 cell line was highest. There was no IgG3 subtype of the anti-diclazuril antibody in any cell line in the present study. Therefore, the antibody of 3D6 2E4was selected to develop the ELISA kit in the follow-up experiments. Antibody specificity refers to the ability to bind to a specific antigen compared with antigen analogs. The cross-reaction rate is commonly used as an evaluation criterion. Cross-reactivity and specificity of an antibody have an inverse relationship. In this study, we determined the specificities for toltrazuril, toltrazuril sulfone, clozaril, monensin, madurmycin, and salinomycin that are similar to diclazuril. These data demonstrated that the mAb was specific for diclazuril and did not show significant cross-reactivity (< 0.01%) with toltrazuril, toltrazuril sulfone, clozaril, monensin, madurmycin, or salinomycin (Table 2).

**TABLE 2:** The cross-reaction rate of diclazuril antibody.

Chemicals	Chemical structural formula	Chemical molecular formula	Cross-reactivity
Diclazuril		C_17_H_9_C_l3_N_4_O_2_	100%
	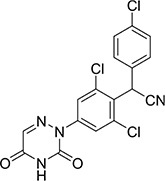		
Toltrazuril		C_18_H_14_F_3_N_3_O_4_S	< 1%
	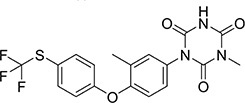		
Toltrazuril 18 sulfone		C_18_H_14_F_3_N_3_O_6_S	< 1%
	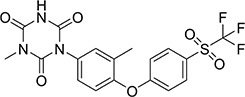		
Clozaril		C_18_H_19_ClN_4_	< 1%
	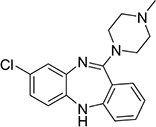		
Monensin		C_36_H_61_NaO_11_	< 1%
	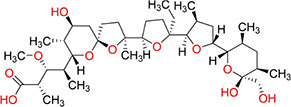		
Madurmycin		C_47_H_83_NO_17_	< 1%
	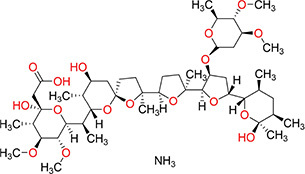		
Salinomycin		C_42_H_70_O_11_	< 1%
	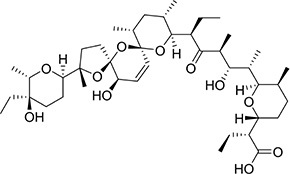		

### Enzyme-Linked Immunosorbent Assay Parameters

**FIGURE 4:**
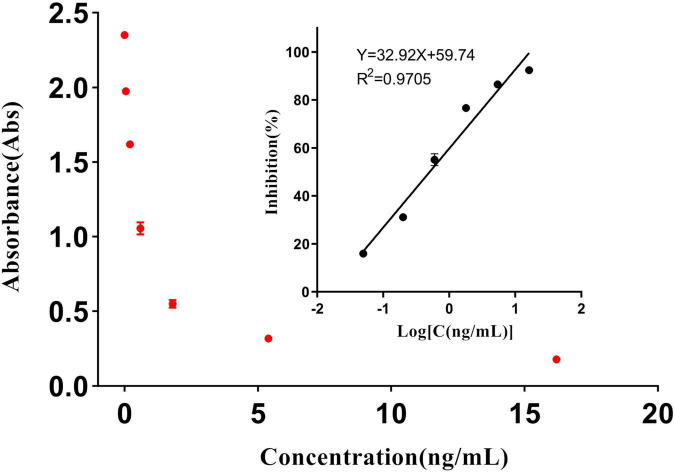
Standard curve of the diclazuril ELISA.

Figure 4 shows a good linear relationship between inhibition and the logarithm of the diclazuril concentration in the range of 0.05–16.2 ng/mL. The linear equation of fitting regression was y = 32.92x + 59.74, the correlation coefficient r was 0.9638, and the IC_50_ value was 0.494 ng/mL. The absorbance of the sample was determined by the same method, and the diclazuril concentration in each sample was calculated from the standard curve.

The sensitivity of the ELISA was evaluated by the IC_50_. Five standard curves were constructed and the IC_50_ value of each curve was calculated. The average IC_50_ value of diclazuril in the ELISA was 0.494 ng/mL and the floating range was 0.449–0.517 ng/mL. The detection limit of the ELISA using this antibody was 0.05 ng/mL. The average Spiked recovery rates of 20 chicken and duck samples ranged from 80 to 110% and the intra- and inter-batch relative standard deviations were < 15% (Table 3). The detection limits of diclazuril in chicken and duck tissues were 0.0963 and 0.0902 ng/mL (Table 4), respectively. To ensure accuracy and stability of the ELISA as well as avoid false positives and negatives, the detection limit of the diclazuril ELISA in poultry tissue samples was set to 0.1 ng/mL.

**TABLE 3:** Determination of detection limits (unit: ng/mL).

Chicken								

**Sample number**	1	2	3	4	5	6	7	8
Determination value	0.07	0.04	0.06	0.05	0.08	0.05	0.05	0.07
Sample number	9	10	11	12	13	14	15	16
Determination value	0.09	0.05	0.06	0.07	0.05	0.05	0.05	0.06
Sample number	17	18	19	20	Average value	Standard deviation	Limit of detection	
Determination value	0.04	0.07	0.05	0.06	0.0586	0.0125	0.0963	

**Duck**								

**Sample number**	**1**	**2**	**3**	**4**	**5**	**6**	**7**	**8**

Determination value	0.06	0.04	0.01	0.06	0.03	0.05	0.06	0.05
Sample number	9	10	11	12	13	14	15	16
Determination value	0.03	0.03	0.03	0.05	0.04	0.01	0.01	0.05
Sample number	17	18	19	20	Average value	Standard deviation	Limit of detection	
Determination value	0.05	0.05	0.04	0.01	0.0380	0.0174	0.0902	

**TABLE 4:** Recoveries and RSD of diclazuril from chicken and duck samples.

Samples	Spiking concentration (μ g/kg)	Kit batch	Recoveries(%)	Intra-batch RSD(*n* = 4)(%)	Average intra-batch RSD(%)	Nter-batch RSD(*n* = 3)%
Duck	0.2	E-1	106.2/102.7/88.1/104.2	8.2	6.3	11.4
		E-2	94.8/83.2/95.2/90.4	6.2		
		E-3	75.2/79.5/81.8/83.1	4.4		
	0.4	E-1	116.2/119.4/105.4/109.6	5.6	7.1	10.7
		E-2	81.2/109.7/99.7/86.9	13.5		
		E-3	101.8/102.5/97.5/100.2	2.2		
	0.8	E-1	104.7/89.8/95.8/8/8.0	8	5.9	9.2
		E-2	78.7/82.5/89.3/79.3	5.9		
		E-3	78.9/85.6/82.8/79.7	3.8		
Chicken	0.2	E-1	86.0/78.3/76.5/75.0	79	8	13.1
		E-2	85.9/79.4/78.3/76.6	80.1		
		E-3	100.0/79.4/100.9/107.6	97		
	0.4	E-1	91.8/79.3/89.5/78.7	84.8	6.7	9.9
		E-2	92.4/104.5/102.2/101.3	100.1		
		E-3	95.6/104.8/93.1/106.9	100.1		
	0.8	E-1	104.2/117.1/93.5/102.6	104.4	8.5	12.6
		E-2	80.7/77.1/93.5/85.9	84.3		
		E-3	102.4/93.8/113.2/105.6	103.8		

The storage condition of the ELISA was 2–8°C. After 12 months, the maximum absorbance value (zero standard), IC_50_ value, and variation in the actual measured value of diclazuril were within 3%. Considering abnormal preservation conditions during transportation and use, the ELISA was stored at 37°C for 7 days. Subsequently, all indexes of the ELISA fully met all requirements. The ELISA was also stored at –20°C for 7 days and the results showed that all index variations were within 3%. Therefore, the ELISA could be stored at 2–8°C for at least 12 months.

### Comparison With HPLC-MS/MS

The results of the 10 negative chicken and duck samples determined by the ELISA and HPLC-MS/MS were negative, and those of the 10 positive chicken and duck samples detected by the ELISA and HPLC-MS/MS were positive. The recovery rates of HPLC-MS/MS and the ELISA were 86.53–115.40% and 81.46–113.52%, respectively. The recheck rate of positive results determined by the ELISA and HPLC-MS/MS was 100%.

### Sequencing Alignment and Analysis

Total RNA extraction from hybridoma cells was successful. RNA quality was assessed by agarose gel electrophoresis. The purity of the RT-PCR products derived from the VH and VL domains of the mAb was sufficient for sequencing.

The VH domain sequence was analyzed using IMGT/V-QUEST and had the following complementarity determining region (CDR) sequences: GFTFSRY (CDR1), SRGGS (CDR2), and GDDNYAFAY (CDR3). The VH domain of the mAb was encoded by an IGHV3 family gene. The VH domain had a predicted molecular weight of 13,273 Da and encoded a protein of 117 residues. The CDR1, CDR2, and CDR3 loops were formed by residues 31–35, 50–65, and 98–106, respectively.

The VH domain nucleotide sequence was GATGTGAAGCTTCAGGAGTCTGGGGGAGGCTTAGTGAAG CCTGGAGGGTCCCTGAAACTCTCCTGTGCAGCCTCTGGAT TCACTTTCAGTAGGTATGCCATGTCTTGGGTTCGCCAGAC TCCAGAGAAGAGGCTGGAGTGGGTCGCATCCATTAGTCG TGGTGGTAGCACCTACTATCCAGACAGTGTGAAGGGCCG ATTCACCATCTCCAGAGATAATGCCAGGAACATCGTGTAC CTGCAAATGACCAGTCTGAGGTCTGAGGACACGGCCATG TATTACTGTGCAAGAGGCGATGATAACTACGCGTTTGCT TACTGGGGCCAAGGGACTCTGGTCACTGTCTCTGCA. The amino acid sequence encoded by mAb 3D6 2E4VH was DVKLQESGGGLVKPGGSLKLSCAASGFTFSRYAMSWVRQTP EKRLEWVASISRGGSTYYPDSVKGRFTISRDNARNIVYLQM TSLRSEDTAMYYCARGDDNYAFAYWGQGTLVTVSA. The VL domain sequence was analyzed using IMGT/V-QUEST and had the following CDR sequences: KSSQSLLNSRTRKNYLA (CDR1), WASTRES (CDR2), and KQSYNLHT (CDR3). The VL domain of the mAb was encoded by an IGKV1 gene. The VL had a predicted molecular weight of 12,297 Da and encoded a protein of 113 residues. The CDR1, CDR2, and CDR3 loops were formed by residues 24–40, 56–62, and 95–102, respectively. The VL nucleotide sequence was GACATCCAGCTGACTCAGTCTCCATCCTCCCTGGCTGTG TCAGCAGGAGAGAAGGTCACTATGAGCTGCAAATCCAG TCAGAGTCTGCTCAACAGTAGAACCCGAAAGAACTACT TGGCTTGGTACCAGCAGAAACCAGGGCAGTCTCCTAAA CTGCTGATCTACTGGGCATCCACTAGGGAATCTGGGGTC CCTGATCGCTTCACAGGCAGTGGATCTGGGACAGATTT CACTCTCACCATCAGCAGTGTGCAGGCTGAAGACCTGG CAGTTTATTACTGCAAGCAATCTTATAATCTTCACACGTT CGGAGGGGGGACCAAGCTGGAAA-TAAAACGG. The amino acid sequence encoded by mAb 3D6 2E4VL was DIQLTQSPSSLAVSAGEKVTMSCKSSQSLLNSR-TRKNYLAW YQQKPGQSPKLLIYWASTRESGVPDRFTGSGSGTDFTLTISS VQAEDLAVYYCKQSYNLHTFGGGTKLEIKR.

### Comparisons With Other Studies and What the Current Study Adds to Existing Knowledge

As an effective, broad-spectrum, and low toxicity anti-coccidiosis drug, diclazuril is widely used, especially in the process of poultry breeding. It is also approved to be used as a feed additive. The residual risk of diclazuril in poultry has gradually attracted attention. Many reports have investigated detection methods of diclazuril residues in animal food. Among the detection methods, there are large instrument detection methods such as high-performance liquid chromatography (HPLC), gas chromatography mass spectrometry, liquid chromatography-tandem mass spectrometry, and high-performance liquid chromatography-mass spectrometry (HPLC-MS). To meet the needs of on-the-spot, rapid, low-cost, and accurate detection, immunological detection methods have become the main research focus. The existing reports of immunological detection methods include gold nanoparticle-based strips (25), gold nanoparticle-based lateral-flow strips (29, 30), enzyme-linked immunoassays (24), and high efficiency chemiluminescent immunoassays (15). The accuracy and stability of immunological methods mainly depend on the antibody sensitivity. The preparation process of antibodies is tedious, and stability between batches is uncontrollable, which is the main deficiency in the application of immunological methods. Therefore, in this study, highly sensitive antibodies were prepared by optimizing the method of hapten modification. The enzyme-linked immunosorbent assay and test kit were developed by optimizing the enzyme-linked immunosorbent assay system. Finally, the variable region of the anti-diclazuril antibody used in this method was sequenced and the gene sequence of the variable region of the antigen recognition site was obtained. It was expected that the protein of the recognition site of the anti-diclazuril antibody could be expressed through a recombinant protein expression system such as *E. coli*, insect cell, or mammalian expression systems. A protein expression system has the advantages of rapid proliferation, high expression, easy purification, stability, and low cost, which can effectively compensate for the shortcomings of immunological methods.

## Conclusion

ELISA-based methods have potential applications for the detection of diclazuril residues and the preparation of a suitable mAb against diclazuril is critical for the development of such a method (20). Diclazuril has a molecular weight that is too small to be immunogenic in accordance with the Landsteiner hapten theory. Therefore, coupling with a macromolecular substance is required to stimulate an immune response in animals. In the present study, diclazuril carboxyl derivatives were prepared using diclazuril, carboxymethyl hydroxylamine, and pyrrolidine. Mice were immunized with a diclazuril–protein conjugate and then mice with polyclonal serum titers against diclazuril were identified. The spleens of these mice were collected, and splenocytes were isolated and fused with myeloma cells to obtain hybridomas. We used synthetic diclazuril antigens to generate hybridoma cell lines and mAbs with high titers, good specificity, and no cross-reactivity. The parameters of a potential diagnostic ELISA were established. The standard curve, limit of detection, recovery, and cross-reactivity were examined. The diclazuril ELISA performed well based on various technical indicators. The sensitivity of the diclazuril ELISA was 0.05 ng/mL. A standard curve generated in the range of 0.05–16.2 ng/mL had a linear correlation coefficient value of ≥ 0.99. The average recoveries of diclazuril from chicken and duck samples were in the range from 85.0 to 102.5%, and intra- and inter-assay coefficients of variation were in the range from 5.9 to 8.5% and 9.2 to 12.6%, respectively. However, the stability and accuracy of an ELISA detection method depend heavily on the sensitivity and stability of the antibody, and the preparation of monoclonal antibodies is tedious and unstable. Therefore, the variable region sequences (VH and VL) of the mAb against diclazuril were determined by cloning and sequencing. This is expected to provide scientific and technical support for stable batch anti-diclazuril antibody expression. Our protocol may be applicable to prepare other recombinant antibodies as well as scFvs, humanized antibodies, chimeric antibodies, bispecific antibodies, and single-domain antibodies. Illegal use and failure to comply with the withdrawal period lead to drug residues in animal edible tissues, which have potential risks to human health. Considering the health of consumers, the residue limit requirements and detection standards for agricultural and veterinary drugs residues have been established (31–33). In the past 2 years, the Codex Aliment Commission has established residue limit standards for 3,724 agricultural and veterinary drugs across 185 types of drugs. Our novel diagnostic ELISA using a mAb against diclazuril may drive additional study on the monitoring and detection of agricultural and veterinary drug residues.

## Data Availability Statement

The original contributions presented in the study are included in the article/Supplementary Material, further inquiries can be directed to the corresponding author/s.

## Ethics Statement

The animal study was reviewed and approved by the Hubei Provincial Science Technology Department.

## Author Contributions

GY: conceptualization. HSh: methodology and writing original draft preparation. CL and HSu: validation. WC: data curation. YY: writing review and editing. JM and YZ: supervision. BC: project administration. All authors contributed to the article and approved the submitted version.

## Conflict of Interest

The authors declare that the research was conducted in the absence of any commercial or financial relationships that could be construed as a potential conflict of interest.

## Publisher’s Note

All claims expressed in this article are solely those of the authors and do not necessarily represent those of their affiliated organizations, or those of the publisher, the editors and the reviewers. Any product that may be evaluated in this article, or claim that may be made by its manufacturer, is not guaranteed or endorsed by the publisher.
