# Supplementary material for: Generation and Characterization of an Anti-diclazuril Monoclonal Antibody and Development of a Diagnostic Enzyme-Linked Immunosorbent Assay for Poultry
Source: Front Nutr. 2022 May 16;9:910876. doi: 10.3389/fnut.2022.910876 (PMC9149080; doi:10.3389/fnut.2022.910876)
Supplement: Supplementary file 1 [file Data_Sheet_1.doc]

**Supplementary Materials**

**Hong Shen^1,2^, Chao Li^2^, Han Sun^2^, Wanqin Chen^2^, Bilian Chen^2^, Yu Yi^1^, Jianfeng Mei^1^, Yanlu Zhang^1^, Guoqing Ying^1^***

^1^Zhejiang University of Technology, College of Pharmaceutical Science, Hangzhou, Zhejiang, China

^2^Zhejiang Institute for Food and Drug Control, Biological Inspection Department, Hangzhou, Zhejiang, China

*** Correspondence:**Prof. Guoqing Ying
**bioph@zjut.edu.cn**

1. **RT-PCR amplification of heavy- and light-chain variable region genes of the anti-diclazuril mAb**
   1. **Primer design**

5′- and 3′-end primers were synthesized in the signal region and constant region to amplify the variable region of the diclazuril antibody heavy-chain (VH). The VH primer sequences were:

VH(5’-3’)

| GATGTGAAGCTTCAGGAGTC |
| --- |
| CAGGTGCAGCTGAAGGAGTC |
| CAGGTGCAGCTGAAGCAGTC |
| CAGGTTACTCTGAAAGAGTC |
| GAGGTCCAGCTGCAACAATCT |
| GAGGTCCAGCTGCAGCAGTC |
| CAGGTCCAACTGCAGCAGCCT |

VH (3’-5’)

| TGCAGAGACAGTGACCAGAGT |
| --- |
| TGAGGAGACTGTGAGAGTGGT |
| TGAGGAGACGGTGACTGAGGT |
| TGAGGAGACGGTGACCGTGGT |

5′- and 3′-end primers were synthesized in the signal region and constant region to amplify the variable region of the diclazuril antibody light-chain (VL). The VL primer sequences were:

VL(5’-3’)

| GATGTTTTGATGACCCAAACT |
| --- |
| GATATTGTGATGACGCAGGCT |
| GATATTGTGATAACCCAG |
| GACATTGTGCTGACCCAATCT |
| GACATTGTGATGACCCAGTCT |
| GATATTGTGCTAACTCAGTCT |
| GATATCCAGATGACTCAGTCT |
| GACATCCAGCTGACTCAGTCT |
| CAAATTGTTCTCACCCAGTCT |

VL(3’-5’)

| CCGTTTCAGCTCCAGCTTG |
| --- |
| CCGTTTTATTTCCAGCTTGGT |
| CCGTTTTATTTCCAACTTTG |
| GGATACAGTTGGTGCAGCATC |

- 1. **Extraction of total RNA from hybridoma cells**

Hybridoma cells in logarithmic growth stage were collected (2×10^6^ cells) and centrifuged at 800 rpm for 5 min. The supernatant was removed and the precipitated cells were stored at - 80℃ or used directly for RNA extraction. RTL solution (350 μL) was added to each cell sample and the mixture was vortexed for 30 s. Ethanol (70%) was added, and the mixture was gently blown evenly with a 1 mL pipette gun. The mixture was transferred to an RNeasy spin column, centrifuged at 8000 × g for 15 s, and the filtrate was removed. RW1 solution (700 μL) was added to the above RNeasy spin column, which was centrifuged at 8000 × g for 15 s, and the filtrate was removed. RPE solution (500 μL) was added to the above RNeasy spin column, which was centrifuged at 8000 × g for 15 s, and the filtrate was removed. RPE solution (500 μL) was added to the above RNeasy spin column, which was centrifuged at 8000 × g for 2 min, and the filtrate was removed. The RNeasy spin column was then placed in a 2 mL collection tube without RNase and centrifuged at full speed for 1 min. Finally, the RNeasy spin column was placed in a 1.5 mL Eppendorf tube without RNase, 30–50 μL H2O without RNase were added to the column, and the tube was centrifuged at 8000 × g for 1 min to elute the RNA.

- 1. **Reverse transcription PCR**

Using the total RNA of hybridoma cells as the template, the SuperScript III One-Step RT-PCR System (Invitrogen, product no.18080-051) was used to reverse transcribe cDNAs.

- 1. **Antibody variable region-specific-primer PCR**

The reaction system and reaction conditions as shown in Tables S1 and S2, the antibody heavy-chain and light-chain variable regions were cloned with specific primers using the cDNA obtained by reverse transcription as the template.

Table S1. The PCR reaction system

| Component | Volume (μL) |
| --- | --- |
| H_2_O | 37.4 |
| 10×pfu buffer（Mg^2+^） | 5 |
| dNTP(10 mM） | 1 |
| pfu | 0.4 |
| Taq | 0.2 |
| Sense primer | 1 |
| Anti-sense primer | 1 |
| cDNA | 4 |

Table S2. The PCR reaction conditions

| Temperature(℃) | Time(min) | Number of cycles |
| --- | --- | --- |
| 94 | 3 | 1 |
| 94 | 1 | 40 |
| 60 | 1 |
| 72 | 2 |
| 72 | 6 | 1 |
| 20 | 60 | 1 |

1. **PCR product cloning and sequencing**

After recovering the nucleotide fragments encoding the antibody heavy-chain and light-chain variable regions, they were cloned into sequencing vectors and sent for DNA sequencing.

- 1. **Gel recovery of PCR products**

PCR products were electrophoresed on 1% agarose gels, and the target strips were recovered by tapping, and by using a Gel Recovery Kit (Generay, GK-2042).

- 1. **Enzyme digestion**

The enzyme digestion system is shown in Tables S3 and S4, the enzyme digestion conditions is 37°C for 30 min, then 85°C for 5 min.

Table S3. Reaction conditions for enzyme digestion

| Component | Volume (μL) |
| --- | --- |
| Plasmid | x (1 μg) |
| 10×FD Buffer | 5 |
| enzyme 1 | 1 |
| enzyme 2 | 1 |
| H_2_O | 43-x |

Table S4. Reaction conditions for enzyme digestion

| Component | Volume (μL) |
| --- | --- |
| Antibody target fragment | 17 |
| 10×FD Buffer | 2 |
| enzyme 1 | 0.5 |
| enzyme 2 | 0.5 |

The fragments from the plasmid enzyme digestion system were recovered by glue running, and the target antibody encoding fragments from the enzyme digestion system were used directly for connection.

- 1. **Connection**

The connection system is shown in Table S5.

Table S5. Reaction conditions for connection

| Component | Volume (μL) |
| --- | --- |
| Carrier glue recovery large fragment | 1.5 |
| Antibody target fragment | 7 |
| 10×ligase buffer | 1 |
| ligase | 0.5 |

- 1. **Conversion**

*Escherichia coli* DH5α competent cells (100 μL) were added to the connection system, placed in an ice-bath for 30 min, heat-shocked at 42°C for 90 s, then placed in an ice-bath for 3 min. Then, 500 μL of LB medium were added and the cells were recovered at 37°C, 120 rpm, for 50 min. The mixture was centrifuged at 4000 rpm for 2 min, most of the supernatant was removed, and the cells were resuspended in the remaining 100 μL of supernatant. The cells were spread on an LB-agar plate. After the plate dried, it was inverted and cultured at 37°C for 12–16 h.

- 1. **Colony PCR identification**

Select 4–6 monoclonal antibodies on each transformation plate and point them to the corresponding resistant LB plate for colony PCR identification. The PCR reaction products were electrophoresed on 1% agarose gels to identify those containing the target fragment. The colony PCR system is shown in Tables S6 and S7.

Table S6. The colony PCR reaction system

| Component | Volume (μL) |
| --- | --- |
| H_2_O | 15.8 |
| 10×pfu buffer（Mg^2+^） | 2 |
| dNTP(10 mM） | 1 |
| Taq | 0.2 |
| Sense primer（20 μM） | 0.5 |
| Anti-sense primer（20 μM） | 0.5 |
| Clone | 1 |

Table S7. The colony PCR reaction conditions

| Temperature(℃) | Time(min) | Number of cycles |
| --- | --- | --- |
| 94 | 3 | 1 |
| 94 | 1 | 25 |
| 60 | 1 |
| 72 | 2 |
| 72 | 6 | 1 |
| 20 | 60 | 1 |

- 1. **Plasmid Extraction**

Colonies found to be positive by colony PCR were selected and the cells were preserved in 20% glycerol. Plasmids were extracted using a Plasmid Miniprep Kit (Biomiga, product no. PD1211-02).

- 1. **Restriction digestion**

The extracted plasmid DNA was verified by restriction enzyme digestion. The system is shown in Table S8. The enzyme digestion conditions is 37°C for 30 min, then 85°C for 5 min. Agarose (1%) gel electrophoresis was used to identify the product bands.

Table S8. Reaction conditions for restriction enzyme digestion

| Component | Volume (μL) |
| --- | --- |
| Plasmid | x (500 ng) |
| 10×FD Buffer | 2 |
| enzyme 1 | 0.5 |
| enzyme 2 | 0.5 |
| H_2_O | 17-x |

- 1. **PCR verification**

The target bands in plasmids identified as positive by restriction digestion were confirmed by PCR. PCR products were electrophoresed on 1% agarose gels. If positive, bands were sent for DNA sequencing with the sequencing primers:

5’ - M13R: CAGGAAACAGCTATGACC

3’ - M13F: TGTAAAACGACGGCCAGT

1. **Sequencing comparison and structural analysis of VH chain and VL chain**

Kabat analysis of the VH chain:

CDR1：RYAMS

CDR2：SISRGGSTYYPDSVKG

CDR3：GDDNYAFAY

Kabat analysis of the VL chain:

CDR1：KSSQSLLNSRTRKNYLA

CDR2：WASTRES

CDR3：FGGGTKLEIKR

The antibodies belong to kappa light-chain subgroup I.

The VH domain has a predicted molecular weight of 13,273 Da (a protein of 117 residues). The CDR1, CDR2, and CDR3 loops are formed by residues 31–35, 50–65, and 98–106, respectively.

The VL domain has a predicted molecular weight of 12,297 Da (a protein of 113 residues). The CDR1, CDR2, and CDR3 loops are formed by residues 24–40, 56–62, and 95–102, respectively.

The nucleotide sequence encoding VH:

GATGTGAAGCTTCAGGAGTCTGGGGGAGGCTTAGTGAAGCCTGGAGGGTCCCTGAAACTCTCCTGTGCAGCCTCTGGATTCACTTTCAGTAGGTATGCCATGTCTTGGGTTCGCCAGACTCCAGAGAAGAGGCTGGAGTGGGTCGCATCCATTAGTCGTGGTGGTAGCACCTACTATCCAGACAGTGTGAAGGGCCGATTCACCATCTCCAGAGATAATGCCAGGAACATCGTGTACCTGCAAATGACCAGTCTGAGGTCTGAGGACACGGCCATGTATTACTGTGCAAGAGGCGATGATAACTACGCGTTTGCTTACTGGGGCCAAGGGACTCTGGTCACTGTCTCTGCA

The amino acid sequence of VH:

DVKLQESGGGLVKPGGSLKLSCAASGFTFSRYAMSWVRQTPEKRLEWVASISRGGSTYYPDSVKGRFTISRDNARNIVYLQMTSLRSEDTAMYYCARGDDNYAFAYWGQGTLVTVSA

The nucleotide sequence encoding VL:

GACATCCAGCTGACTCAGTCTCCATCCTCCCTGGCTGTGTCAGCAGGAGAGAAGGTCACTATGAGCTGCAAATCCAGTCAGAGTCTGCTCAACAGTAGAACCCGAAAGAACTACTTGGCTTGGTACCAGCAGAAACCAGGGCAGTCTCCTAAACTGCTGATCTACTGGGCATCCACTAGGGAATCTGGGGTCCCTGATCGCTTCACAGGCAGTGGATCTGGGACAGATTTCACTCTCACCATCAGCAGTGTGCAGGCTGAAGACCTGGCAGTTTATTACTGCAAGCAATCTTATAATCTTCACACGTTCGGAGGGGGGACCAAGCTGGAAATAAAACGG

The amino acid sequence of VL:

DIQLTQSPSSLAVSAGEKVTMSCKSSQSLLNSRTRKNYLAWYQQKPGQSPKLLIYWASTRESGVPDRFTGSGSGTDFTLTISSVQAEDLAVYYCKQSYNLHTFGGGTKLEIKR

1. **Detection conditions for diclazuril by HPLC-MS/MS**
   1. Preparation of solutions

Stock standard solution: Standard (10 mg) was accurately weighed, dissolved in 50 mL methanol, and mixed to homogeneity. The concentration of the resulting solution was 200 µg/mL. The solution was stored at <−18°C in the dark for <12 months.

Mixed standard solution: Stock standard solution (0.5–10 mL) was accurately measured into a volumetric flask, diluted with methanol to 10 mL, and mixed to homogeneity. The concentration of the resulting solution was 10 µg/mL. The solution was stored at <−18°C in the dark for <6 months.

Standard working solution: An appropriate volume of mixed standard solution was accurately measured and diluted to a suitable concentration with methanol:water (80:20 v/v) just before use.

Stock internal standard solution: Internal standard (10 mg) was accurately weighed, dissolved in 50 mL methanol, and mixed to homogeneity. The concentration of the solution was 200 µg/mL. The solution was stored at <−18°C in the dark for <12 months.

Internal standard working solution: Internal stock standard solution (0.5–10 mL) was accurately measured into a volumetric flask, diluted with methanol to 10 mL, and mixed to homogeneity. The concentration of the resulting solution was 10 µg/mL. The solution was stored at <−18°C in the dark for <6 months.

- 1. Sample preparation

Sample (5.0 g) was accurately weighed into a 50 mL centrifuge tube. Then, 50 μL internal standard working solution was added and the sample was vortexed for 30 s. Anhydrous sodium sulfate (5 g) and 25 mL ethyl acetate were added to the sample, which was homogenized for 1 min, and then the mixture was centrifuged at 3000 rpm for 10 min. Supernatant (5 mL) was transferred into a 15 mL glass tube and evaporated to dryness at 45°C under a gentle stream of nitrogen. The residue was dissolved in 10 mL of a mixture of cyclohexane and ethyl acetate (1:1 v/v). Finally, the extract was purified by GPC and analyzed by LC-MS/MS.

- 1. HPLC conditions:

1. Column: ACQUITY UPLC BEH C_18_ column (100 × 2.1 mm, 1.7 µm).
2. Mobile phase: 45% A: 0.05% acetic acid solution; 55% B: acetonitrile.
3. Flow rate: 400 μL /min.
4. Column temperature: 30 ℃.
5. Injection volume: 10 μL.
   1. The MS conditions:
6. Sheath gas: 30 U.
7. Auxiliary gas: 8 U.
8. Ion spay voltage in ESI-mode: -3500 V.
9. Capillary temperature: 320℃.
10. Source CID: 10 V.
11. Width of Q1 and Q3: 0.7.
12. Collision gas: high-purity argon.
13. Collision gas pressure: 1.5 m Torr.
14. Retention time: 3.88 min.
15. Ion pairs: 405.1/299.0 and 405.1/334.1.
16. Collision energy:19 ev, 19 ev.
17. Instrument: ACQUITY/Xevo- TQ-S (Waters, Milford, USA).
    1. Quantitative analysis

We selected standard working solution that gave a similar response to that of the test sample solution. The responses of the analyte in the standard working solution and the sample solution were within the linear range of the instrument detection. Mixed standard working solution and sample solution were injected alternately with equal injection volume.

- 1. Determination of dynamic range and the limit of quantification (LOQ)

A standard curve was established for diclazuril over the concentration range of 0-50 ng/mL (0, 0.5 ng/mL, 2.5 ng/mL, 5 ng/mL, 10 ng/mL, 25 ng/mL and 50 ng/mL). The regression equation for the relationship between the concentration and the response was y=89.73x + 71.51 (R^2^=0.9955). The limit of quantification (LOQ) was estimated by analyzing the standard solutions. We defined the LOQ as the diclazuril concentration in the samples that produced a S/N ratio of 10. The limit of quantification for diclazuril was 1 μg/kg.


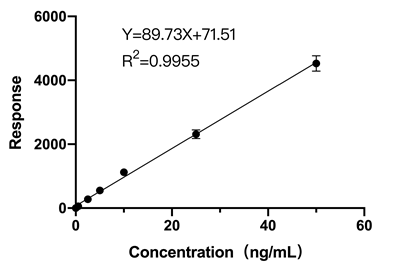


Fig. S1. Standard curve for diclazuril by HPLC-MS/MS

- 1. Spiked recovery tests

Diclazuril was spiked into chicken and duck samples at 5, 10, and 25 μg/kg to evaluate the feasibility of using LC-MS/MS to detect diclazuril in real poultry. The test results are shown in Table S9. The mean diclazuril recoveries from the spiked poultry were 85.46%–112.24%, and the relative standard deviation was 1.56%–6.36%.

Table S9. Recovery and relative standard deviation of diclazuril residue in chicken and duck samples (n = 3)

|  | Sample | Spiked concentration (μg/kg） | LC-MS/MS | |
| --- | --- | --- | --- | --- |
|  |  |  | Recovery (%) | RSD (%) |
| Diclazuril | chicken | 5 | 85.46 | 1.79 |
|  |  | 10 | 91.53 | 3.14 |
|  |  | 25 | 112.24 | 1.56 |
|  | duck | 5 | 88.58 | 6.36 |
|  |  | 10 | 89.24 | 5.72 |
|  |  | 25 | 110.57 | 2.48 |
